# Optimization of oviposition trap settings to monitor populations of *Aedes* mosquitoes, vectors of arboviruses in La Reunion

**DOI:** 10.1038/s41598-022-23137-5

**Published:** 2022-11-02

**Authors:** Ronan Brouazin, Iris Claudel, Renaud Lancelot, Guillaume Dupuy, Louis-Clément Gouagna, Marlène Dupraz, Thierry Baldet, Jérémy Bouyer

**Affiliations:** 1 UMR Mivegec (Maladies Infectieuses et Vecteurs: Écologie, Génétique, Évolution et Contrôle), IRD-CNRS-Univ. Montpellier, 97410 Saint-Pierre, La Réunion France; 2 UMR Astre (Animals, Health, Territories, Risks, Ecosystems), Cirad, Inrae, Univ. Montpellier, 34398 Montpellier, France; 3 Cirad, UMR Astre, 97491 Sainte Clotilde, La Réunion France; 4 ARS Réunion, Service de Lutte Anti-Vectorielle, Saint-Denis, La Réunion France; 5 UMR Mivegec, 34394 Montpellier, France; 6 Insect Pest Control Laboratory, Joint FAO/IAEA Programme of Nuclear Techniques in Food and Agriculture, IAEA Vienna, Wagramer Strasse 5, 1400 Vienna, Austria

**Keywords:** Ecology, Animal behaviour, Entomology, Epidemiology

## Abstract

Several dengue epidemics recently occurred in La Reunion, an island harboring two dengue viruses (DVs) vectors: *Aedes albopictus*, and *Ae. aegypti*, the former being the main local DV vector. *Aedes aegypti* shows a peculiar ecology, compared to other tropical populations of the same species. This study aimed to provide researchers and public-health users with locally validated oviposition traps (ovitraps) to monitor *Aedes* populations. A field experiment was performed in Saint-Joseph to assess the effect of different settings on the detection probability and apparent density of *Aedes* mosquitoes. Black plastic ovitraps were identified as the best choice. Vacoa trees (*Pandanus utilis*) were the only observed breeding sites for *Ae. aegypti*, shared with *Ae. albopictus*. They were the experimental units in a Latin square design with three factors: trap position in the trees (ground vs canopy), oviposition surface in the trap (blotting paper vs. vacoa leaf), and addition of organic matter to the trap water. The latter factor was found unimportant. On the ground, *Ae. aegypti* eggs were only found with vacoa leaves as the oviposition surface. Their detection and apparent density increased when ovitraps were located in the tree canopy. The main factor for *Ae. albopictus* was the oviposition surface, with a preference for blotting paper. In all trap settings, their detection was close to 100%. Larval survival was lower for a high egg density, combined with blotting paper as the oviposition surface. When monitoring mixed *Aedes* populations in La Reunion, we recommend using black plastic ovitraps, placed at 1.50-to-2.00-m high in vacoa trees, with vacoa leaves as the oviposition surface.

## Introduction

### A changing epidemiology of mosquito-borne viruses

Dengue is the most widely distributed mosquito-borne viral infection. Dengue virus (DV) is primarily transmitted by *Aedes* mosquitoes, with an annual incidence of cases increasing from ca. 29 million in 1990, to ca. 57 million in 2019^1^. DV is prevalent in 128 countries, mostly located in tropical regions, where rainfall, temperature, relative humidity and rapid unplanned urbanization favor DV mosquito vectors^2–7^.

Worldwide, *Aedes aegypti* is the most important and efficient DV vector, followed by *Aedes albopictus*^7,8^. Nevertheless, in several southwestern Indian Ocean islands (SWOIs), especially in La Reunion, *Ae. albopictus* is considered the main DV vector^9^. Several dengue epidemics have been reported since 1977/78, when a large type-2 DV outbreak infected ca. 30% of the population^10^. However, only 3 to 31 sporadic DV cases were reported yearly, until the occurrence of a new type-1 DV outbreak, with 228 reported cases^11^. Since 2015, DV outbreaks have occurred yearly^12^. More than 16,000 cases were reported in 2020^13^. At the end of July 2021—i.e., at the end of DV transmission season, public-health authorities reported 27,213 confirmed dengue cases and 15 deaths as of January 2021^14^. The consecutive circulation of several DV serotypes is accompanied by an increased risk of clinical severity, a possible sign of DV endemization^15^.

In addition to DV, *Aedes albopictus* is also the main vector of the chikungunya virus (CV)^16^. In July 2004, CV first spread from outbreaks on the East African coast to the Comoros Archipelagos, and all the other southwestern Indian Ocean (SWIO) islands thereafter^17^. In La Reunion, CV transmission was intense, with 300,000 cases recorded during a first wave (2005), and more than 250,000 cases recorded during a second wave (2006), with more than 200 deaths, despite much effort devoted to the control of vector populations^18,19^.

### *Aedes aegypti* in La Reunion: uncovering peculiarities

Soghigian et al*.*^20^ found the common ancestor *Ae. aegypti s.s.*, after diverging from *Ae. mascarensis* seven million years ago, colonized the African continent less than 85,000 years ago, long after it had colonized La Reunion and other SWIO islands, including Madagascar. Kotsakiozi et al*.*^21^ reported that the *Ae. aegypti* strain found on La Reunion was genetically unique, and close to *Ae. mascarensis*. Thus, *Ae. aegypti* mosquitoes found nowadays in this island might have diverged from *Ae. mascarensis* populations. This last species is absent from La Reunion, but endemic to Mauritius^22^. Indeed, *Ae. aegypti* mosquitoes described in La Reunion, are peculiar. They show the morphological traits of *Ae. aegypti aegypti*, sharing ecological traits with *Ae. aegypti formosus*. Both mosquitoes are considered subspecies of *Ae. aegypti s.s.* spreading on the African continent less than 1000 years ago^20^. On La Reunion, the distribution of *Ae. aegypti* mosquitoes seems to be restricted to a few rural areas^23^. This tiny distribution is attributed to competition with *Ae. albopictus* mosquitoes, which are predominant in urban areas^24^. The same authors also identified evidence of introgression events from Asian strains of *Ae. aegypti aegypti*, which were probably moped up from urban environments during DDT campaigns against malaria vectors^20^.

Since the first description of *Ae. aegypti* in Saint-Paul, La Reunion in 1907^25^, little information on its preferred development sites was obtained, unlike *Ae. albopictus*. The latter shows a strong ecological plasticity. It is present on almost all along the island’s coastline, up to an altitude of 1,200 m (800 m during the dry season), both in urban and rural sites. It uses natural and anthropogenic water collections such as rock holes and saucers under flower pots as larval sites^23,26^. Following the discovery of *Ae. aegypti* in 1907, a discontinuous distribution up to 600 m altitude, especially in the western part of the island, was observed in the 1950s^27^. Thereafter, observations of *Ae. aegypti* mosquitoes were not reported for more than 30 years: the population seemed to have disappeared from La Reunion. It was actually restricted to a few natural habitats^23^, such as rock holes that it shares with *Ae. albopictus* and is never found in artificial sites^24^, unlike the urban pan-tropical *Ae. aegypti aegypti*. On La Reunion, the distribution of *Ae. aegypti* remains poorly understood. Individuals of this species are rarely caught by the oviposition trap network implemented by the Regional Health Agency (ARS) to monitor *Ae. albopictus* populations. Thus, only a few *Ae. aegypti* eggs vs. thousands of *Ae. albopictus* eggs, were collected by the ARS ovitrap network in the months preceding our study: 5 vs. 4,063 in 15 ovitraps set in our study site from 28 July 2020, to 6 October 2020, G. Dupuy, unpublished data).

This study was the first step of a research project aiming at providing methods and tools for controlling *Aedes* mosquito populations with the boosted Sterile Insect Technique (SIT). This mosquito-control strategy is derived from classical SIT, which consists of large-scale release of sterile male mosquitoes to reduce wild mosquito populations. Mosquitoes are mass-reared in the laboratory, sexed, and then sterilized through a mixture of X- and $\gamma$-ray irradiation before being released in the wild. Wild virgin females mate with sterile males and therefore have no offspring. In boosted SIT, sterile males are coated with a biocide before release. They are used as carriers of biocides to females and larval sites. The boosted SIT approach may allow considerable cost savings by reducing the numbers of sterile males to be released, as well as the frequency of releases^28,29^.

A pilot trial of boosted SIT was conducted in 2021 against *Ae. aegypti* in Saint-Joseph in the south of La Reunion. Accurate knowledge of the ecology and phenology of target mosquito populations is needed before starting any SIT-based control program^30^. For instance, egg sterility—combined with the ratio of released sterile to wild males (available from the outcome of adult traps, such as CO_2_baited BG traps), allows assessing the competitiveness of sterile vs. wild males^31^. In addition, competitive release—i.e., better population dynamics, is expected for *Ae. albopictus* following the population reduction of its competitor, i.e. occupying the same breeding sites. Detecting such changes would require a sensitive trapping method (i.e., detecting *Aedes* mosquitoes where they are present), without overlooking any population. Thus, it was necessary to optimize the sensitivity and efficiency of oviposition traps, a widespread trapping method used to assess the density of eggs laid down by gravid *Aedes* females^32,33^.

**Figure 1:**
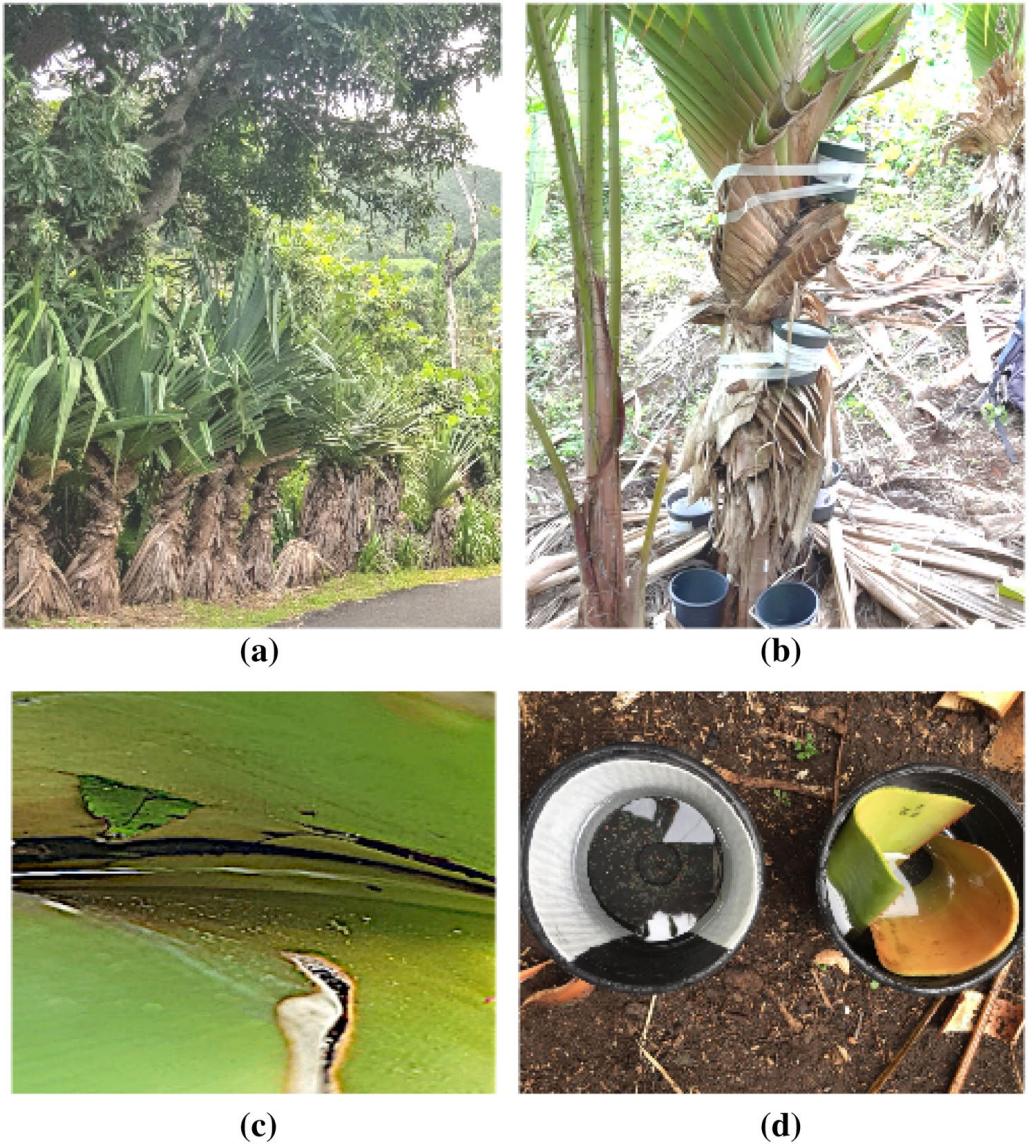
Vacoa trees (*Pandanus utilis*) and oviposition traps used during a field experiment in Saint-Joseph, La Reunion, 2020. (**a**) Vacoa trees along the road in one of the study sites; (**b**) preliminary trial with ovitraps strapped on the trunk of a vacoa tree; (**c**) water accumulation at the inner base of a vacoa leaf; (**d**) two ovitraps, either with blotting paper (left trap), or with vacoa leaf (right trap) as the oviposition surface.

Preliminary observations in the study area revealed rock holes, human-created microhabitats, and tree holes exclusively hosting *Ae. albopictus* larvae. *Aedes aegypti* larvae were only found in water collections at the inner base of vacoa-tree leaves (*Pandanus utilis*) (Fig. 1a–c, and Fig. S3), together with *Ae. albopictus* larvae.

### Goals

The aim of this field experiment was to optimize oviposition trap settings, including height position, baiting scheme and oviposition surface, for the detection of *Aedes* mosquito populations, and assessment of their density.

The main question addressed in this field experiment was as follows:

*What is the best setting for ovitraps - including for the L4 laboratory-rearing step, to maximize the probability to detect Ae. aegypti and Ae. albopictus mosquitoes, and assess the relative apparent density of their populations?*

The study was performed in Saint-Joseph, a municipality located in the south of La Reunion. Two sites were selected after preliminary investigations to assess the presence of well-established *Ae. aegypti* and *Ae. albopictus* mosquito populations:

- a 10-ha tree orchard located along the Langevin River, planted with vacoa trees (*Pandanus utilis*), coconut trees and other indigenous or endemic trees (latitude 21°23′04.2″S, longitude 55°38′45.3″E),
- an isolated “ravine” (i.e., a narrow valley) with a riparian forest, covering ca. two ha, lying between the main road and the Indian Ocean (latitude 21°22′48.2″S, and longitude 55°39′34.7″E) in the Vincendo district of St Joseph.

### Study design

Black plastic ovitraps were used in the field experiment (Fig. 1b): *Ae. aegypti* female mosquitoes prefer to lay their eggs in black containers^32,34–36^. Each ovitrap was filled with 250 mL of tap water. A Latin square design was adopted to control the effect of external factors possibly influencing the outcome: air temperature, relative humidity, wind speed and direction, habitat suitability for feeding, breeding, and resting. At each site, two vacoa trees of two to three meters high were selected and kept throughout the experiment. Three bimodal factors were crossed on each tree (i.e., eight traps/tree) during a given trapping session:

- 1. Height position (covariate *pos*) in the vacoa tree: ground level (“ground” category) vs. tree-canopy level (“canopy” category),
- 2. Oviposition surface (covariate *surf*): blotting paper strip, safety assessed for *Aedes* eggs (“paper” category) vs. piece of vacoa leaf (“leaf” category): see Fig. 1d,
- 3. Organic matter (covariate *om*): addition of organic matter (“added” category), or not (“none” category) to trap water. We used commercial fish food (JBL Novo Cuppy).

With two vacoa trees per site, a total of 32 outcomes were recorded on each trapping session: two sites * two trees / site * eight traps / tree. Each ovitrap had four possible (*surf*, *om*) combinations. Thus, four trapping sessions were needed to complete a replicate, i.e., 128 outcomes/replicate. Each replicate lasted two weeks. Two replicates were implemented in this experiment, for a total of 256 outcomes.

## Results

The study spanned from November 30, 2020 to January 14, 2021. A total of 11,826 eggs were collected from 256 trapping sessions. Identified mosquito fourth-instar larvae (L4) summed up to 6,992, finally splitting into 975 *Ae. aegypti*, and 6,017 *Ae. albopictus* larvae.

### Optimization of ovitrap settings in the field

#### Overview

**Figure 2:**
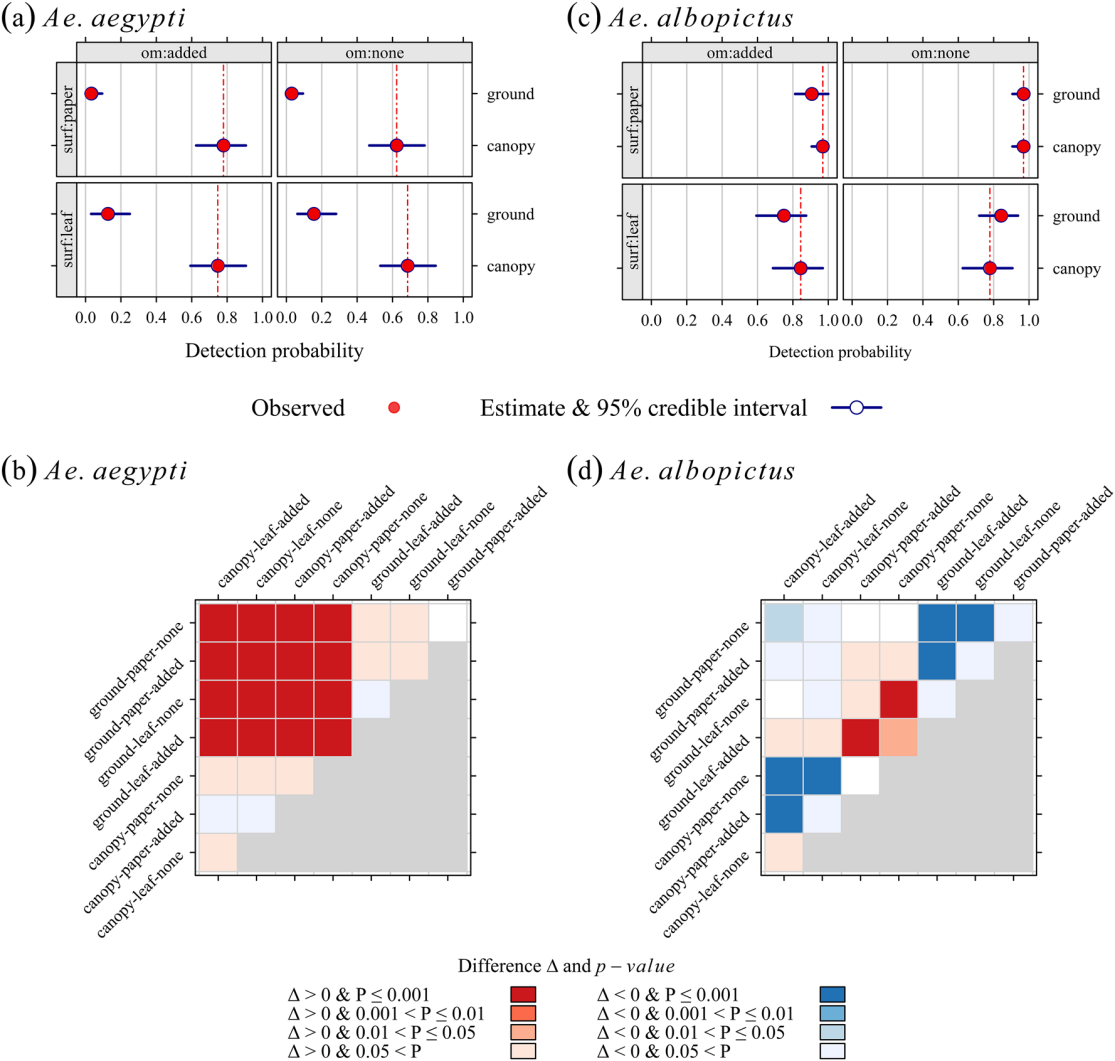
Detection probability of fourth-instar larvae (L4): estimates of population means and their bootstrap 95% credible interval (1000 replicates) for *Aedes aegypti* (**a**), and *Aedes albopictus* (**b**); pairwise tests for the significance of differences Δi,j in estimates (column *j*–row *i*) for *Aedes aegypti* (**c**), and *Aedes albopictus* (**d**); Row and column labels: three-word code as follows) (1) trap position: ground/canopy, (2) oviposition surface: paper/leaf. (3) Organic matter added to trap water: none/added. Models were fitted with data collected during a field experiment in La Reunion, 2020 (256 ovitrap outcomes, 11,826 eggs).

**Figure 3:**
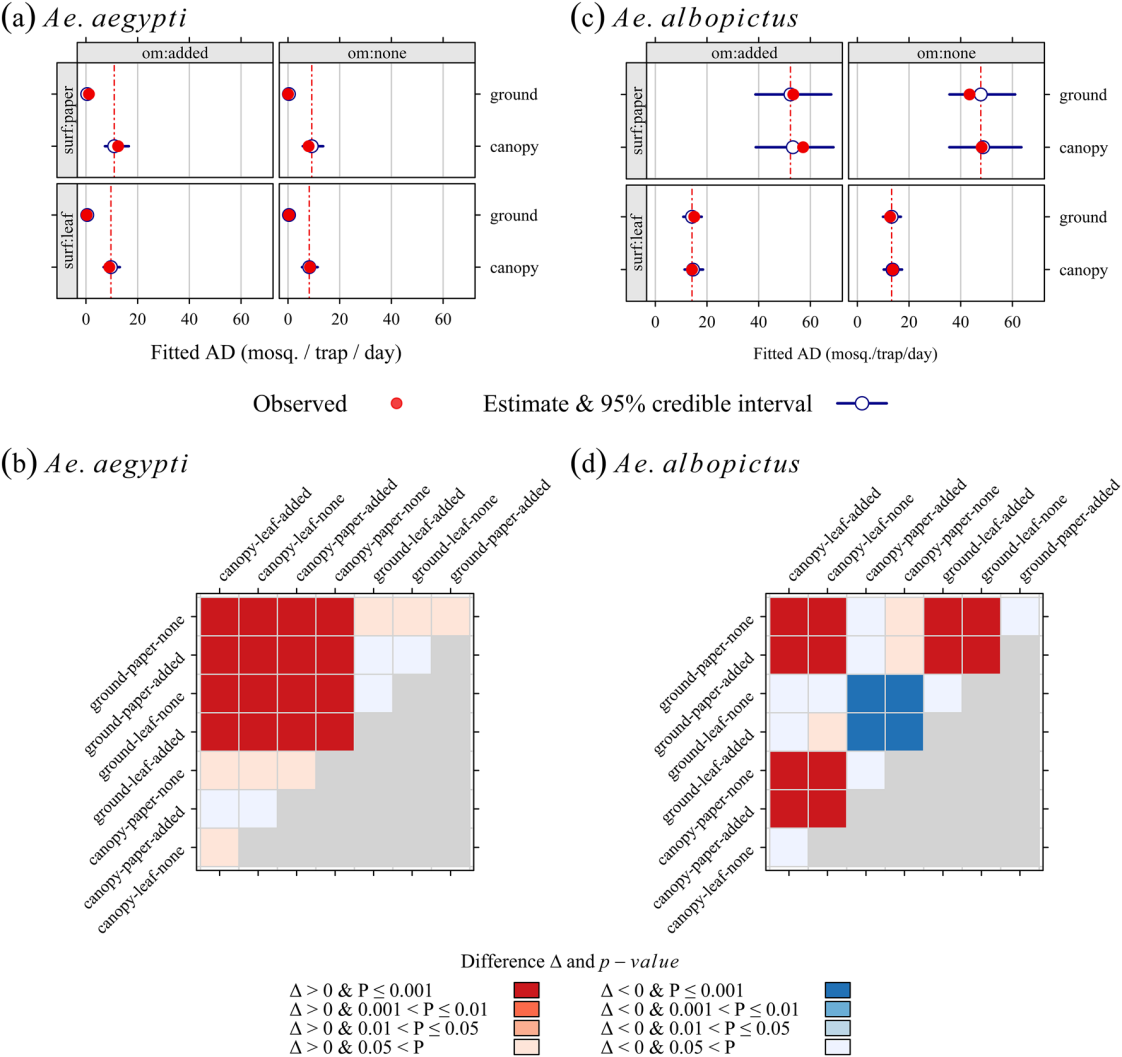
Apparent density of fourth-instar larvae (L4): estimates of population means and their bootstrap 95% credible interval (1000 replicates) for *Aedes aegypti* (**a**), and *Aedes albopictus* (**b**); pairwise tests for the significance of differences Δi,j in estimates (column *j*—row *i*) for *Aedes aegypti* (**c**), and *Aedes albopictus* (**d**); Row and column labels: three-word code as follows) (1) Trap position: ground/canopy, (2) Oviposition surface: paper/leaf. (3) Organic matter added to trap water: none/added. Models were fitted with data collected during a field experiment in La Reunion, 2020 (256 ovitrap outcomes, 11,826 eggs).

A graphical presentation of the main results is provided in Fig. 2 (detection probability) and Fig. 3 (apparent density): population means and pairwise tests for differences in population means (H_0_: no difference in population means). The apparent-density estimates are shown in Tables S1 (*Ae. aegypti*) and S2 (*Ae. albopictus*). Estimates of the MMA model coefficients are available in Tables S3 (*Ae. aegypti*) and S4 (*Ae. albopictus*).

#### Detection probability

##### *Aedes aegypti* mosquitoes

The most important variable in the multimodel averaging (MMA) hurdle model—combining detection probability and apparent density—was the trap position in vacoa trees (Fig. S1). It was selected in 100% of the plausible models kept for the MMA, with each of the 1000 resampled datasets. Its coefficient—in the detection probability submodel—was strongly negative and highly significant (Tables S3: − 3.38 for the position on the ground, P < 0.001). The two other covariates were less important. Their coefficients were small and not significant (P > 0.05, Fig. S1, Table S3).

The detection probability was low when the ovitraps were set on the ground, irrespective of the addition of organic matter to the trap water (Fig. 2a, Table S1):

- The estimate was close to 0 when the oviposition surface was a strip of blotting paper (Fig. 2a). This configuration (ovitrap on the ground + blotting paper strip as the oviposition surface) should not be used to monitor *Ae aegypti* populations in La Reunion.
- The estimate was still less than 20% when the oviposition surface was a piece of vacoa leaf (Fig. 2a).

Symmetrically, the highest detection probability (Fig. 2a: ca. 0.8) was observed for ovitraps lying in the canopy of vacoa trees, with similar values (Fig. 2a: low differences, Fig. 2b: P > 0.05) when the oviposition surface was a blotting paper strip, or a piece of vacoa leaf.

The predominance of height position in vacoa trees—over the two other covariates—is highlighted in Fig. 2b: the differences in detection probability between the tree canopy and the ground level were large (from 50 to 80%), and significant (P < 0.001), regardless of the combination with the other covariates. Hence, the cell block crossing canopy with ground categories is uniformly colored in dark red (P < 001). No other differences than those between traps in the tree canopy, and traps on the ground, were significant (P > 0.05).

##### *Aedes albopictus* mosquitoes

The most important variable in the MMA hurdle model was the oviposition surface (Fig. S1). It was selected in 100% of the plausible models kept for MMA, with each of the 1000 resampled datasets. Its coefficient—in the detection probability submodel, was positive and significant (Table S3: 1.597 for the blotting paper strip coefficient, P = 0.001). The two other covariates were less important. Their coefficients were small and not significant (P > 0.05, Fig. S1, Table S3).

The detection probability of *Ae. albopictus* mosquitoes was always high, regardless of the ovitrap setting. It ranged from ca. 70% for ovitraps lying on the ground, with a piece of vacoa leaf as the oviposition surface, and organic matter added to trap water), to nearly 100% when the ovitraps were set on the canopy of vacoa trees (irrespective of the type of oviposition surface), or when they were set on the ground, with a blotting paper strip as the oviposition surface (Fig. 2c).

For *Ae. albopictus*, the effect of oviposition surface on the detection probability was lower than the effect of height position, in contrast to *Ae. aegypti*.

The effects of covariates on the apparent density (Fig. 3) were similar to those reported for the detection: importance of the height position of ovitraps for *Ae. aegypti*, vs. importance of oviposition surface for *Ae. albopictus* (Fig. S1).

The fitted apparent density (Fig. 3, and Tables S1 for *Ae. aegypti*, and S2 for *Ae. albopictus*) reinforced the detection results.

Setting ovitraps on the ground did not produce robust estimates for *Ae aegypti* mosquito populations—regardless of the other settings. Thus, the fitted apparent density (Table S1) ranged from 0.0 to 1.1 mosq./trap/day (blotting paper as the oviposition surface, without or with organic matter in trap water), and from 0.3 to 0.4 mosq./trap/day (vacoa leaf as the oviposition surface, without or with organic matter in trap water). In comparison, the fitted values for *Ae. albopictus* (Table S2) ranged from 12.7 to 15.1 mosq./trap/day (vacoa leaf as the oviposition surface, without or with organic matter in trap water), and from 43.4 to 53.4 mosq./trap/day (blotting paper as the oviposition surface, without or with organic matter in trap water).

For *Ae aegypti*, setting ovitraps on vacoa canopy significantly increased the observed and fitted values (P < 0.001), w.r.t. the ground level (Fig. 3a,c, Table S1).

For *Ae. albopictus*, the highest estimates were obtained at the canopy level with blotting paper as the oviposition surface, and organic matter in trap water: 57 mosq./trap/day, with a 95% credible interval (CI) [39, 69]. These estimates were similar to those obtained at the ground level—or at the canopy level, with blotting paper as the oviposition surface, without or with organic matter in trap water. Using blotting paper as the oviposition surface was thus the main factor for *Ae. albopictus* apparent density: all pairwise differences were strong and highly significant (P < 0.001) for blotting-paper vs. vacoa-leaf comparisons (Fig. 3b,d, Table S2).

### Proportion of *Aedes aegypti* in the total *Aedes* L4 count

**Figure 4:**
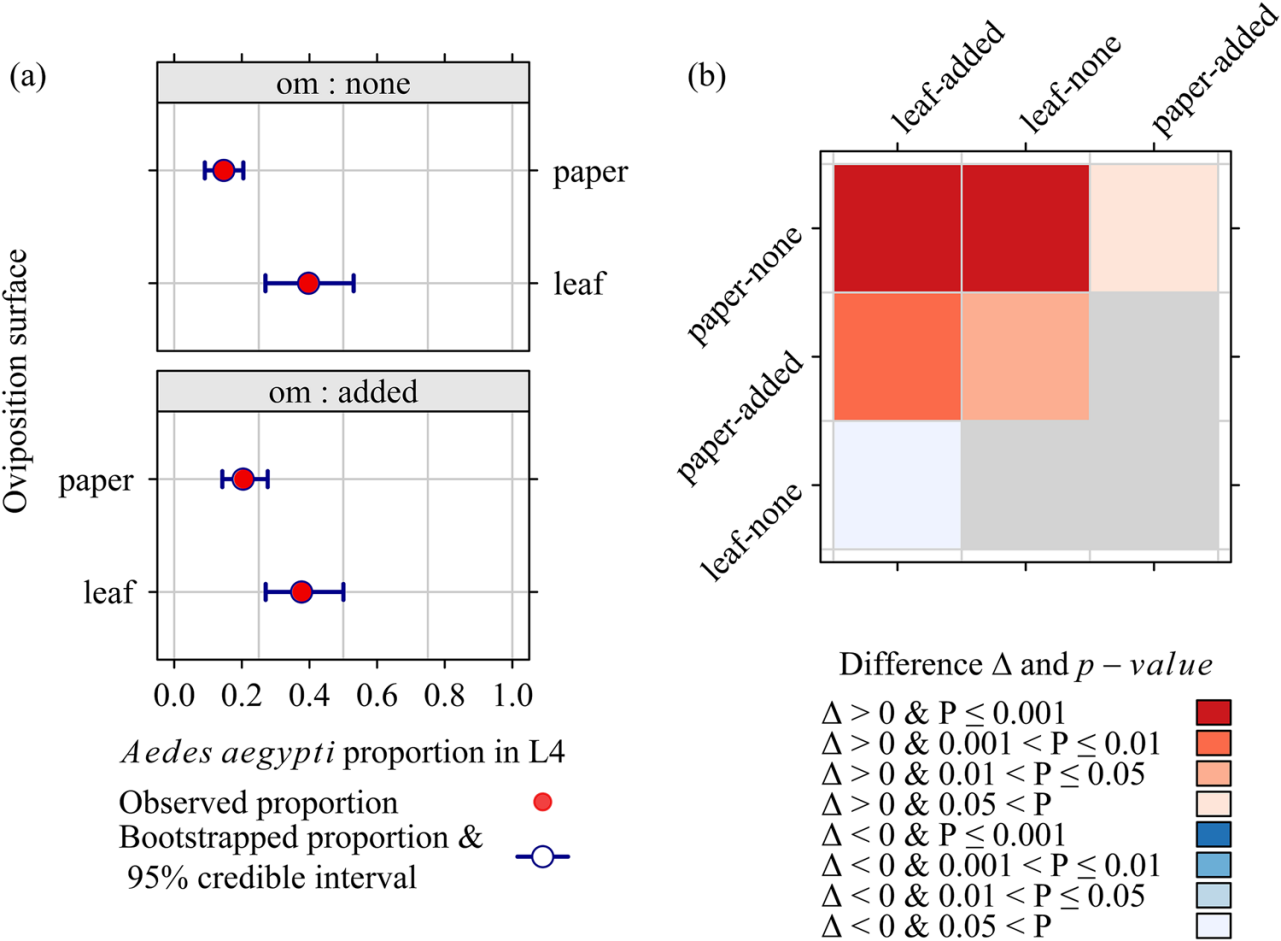
Proportion of *Aedes aegypti* in fourth-instar larve (L4) emerging from *Aedes* eggs collected in oviposition traps set at the canopy of vacoa trees, observed during a field experiment in La Reunion, 2020 (119 ovitrap outcomes, 9277 eggs); (**a**) Estimates and bootstrap 95% credible intervals (1000 replicates), (**b**) pairwise tests for the significance of differences Δi,j in estimates (column *j*–row *i*). Row and column labels: two-word code (1) oviposition surface: paper/leaf. (2) Organic matter added to trap water: none/added.

A specific analysis was performed to select ovitrap settings providing density estimates of *Ae. aegypti* and *Ae. albopictus* similar to those reported in another study concomitantly implemented in the same site^37^. The previous step of the analysis provided evidence that *Ae. aegypti* rarely goes to the ground. Therefore, we discarded data collected at the ground level, and estimated the proportions of *Ae. aegypti* vs. the oviposition surface, and the addition of organic matter to the trap water. Figure 4a,b show that the addition of organic matter had little and nonsignificant (P > 0.05) influence on *Ae. aegypti* proportion in the overall *Aedes* L4 count.

With a blotting paper strip as the oviposition surface, the proportion always remained low: 14% [9, 20], for traps without, and 20% [14, 28] for traps with, organic matter added to the trap water. Conversely, using a piece of vacoa leaf as the oviposition surface provided more accurate estimates: 40% [27, 53] without organic matter, and 38% [27, 50] with organic matter.

### Survival of fourth-instar larvae during the laboratory-rearing step

**Figure 5:**
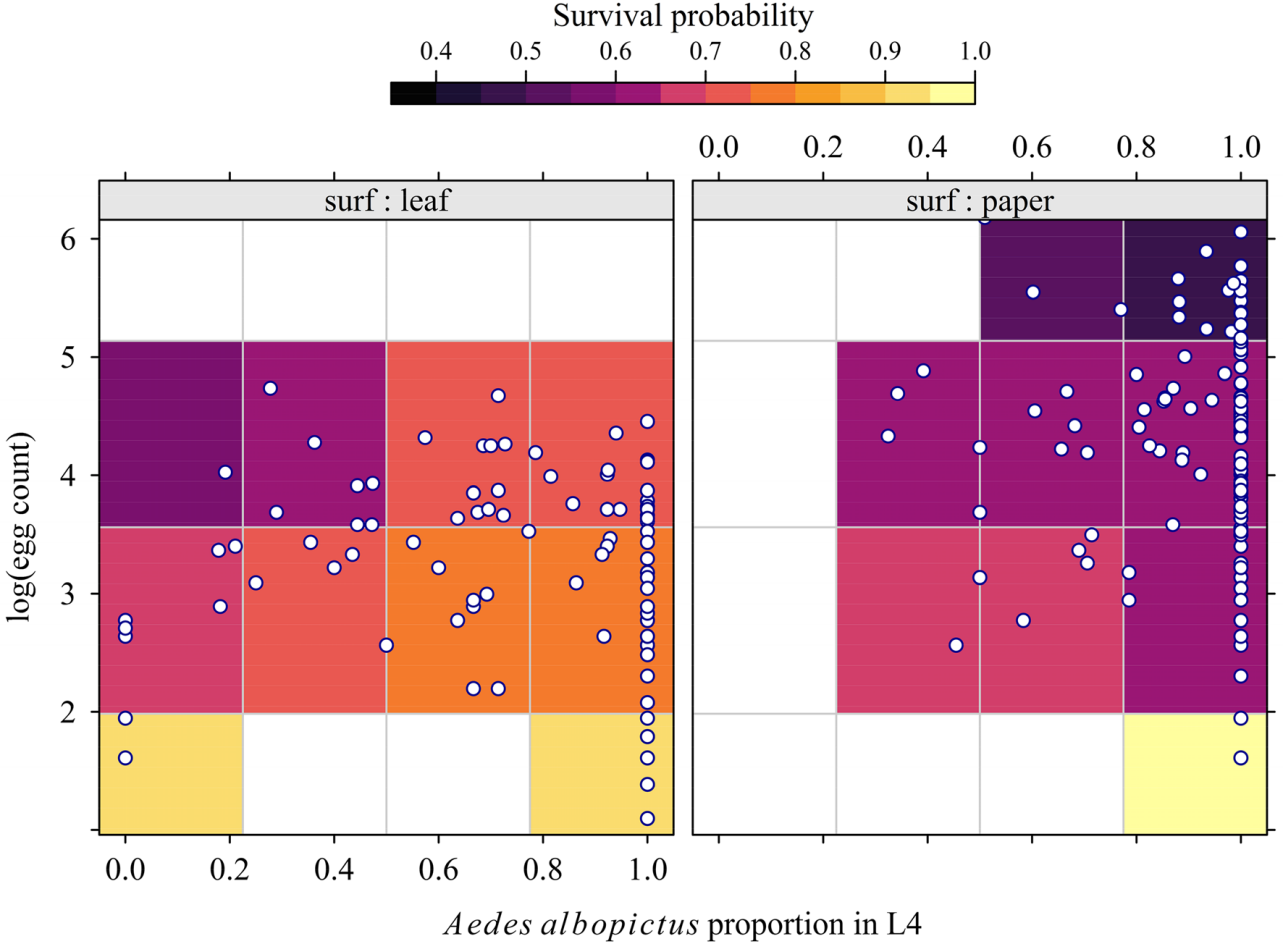
Observed survival of fourth-instar larvae (L4) according to the proportion of *Aedes albopictus* in total L4, the log(apparent density) of *Aedes* eggs, and the oviposition surface. Data were collected during a field experiment in La Reunion, 2020 (256 ovitrap outcomes, 11,826 eggs).

Among the 11,826 collected eggs, the overall survival at the L4 stage was 59.1%. The “non survivors” were either non-hatched eggs (hatch rate $\simeq$ 80%), or larvae dying before being diagnosed and counted, mainly because of cannibalism. Figure 5 gives an overview of the data (232 trapping outcome). The survival of L4 is represented by the colored level plot: the darker the plot regions, the lower the survival. The observed survival ranged from 0.12 to 1.00 (mean = 0.68, median = 0.70). The lowest survival occurred in the right panel (eggs collected on blotting paper strips as the oviposition surface), more precisely in the upper right region of this plot, where the egg density ($E$ ranging from 2 to 619, mean = 69, median = 41) was high, as was the proportion of *Ae. albopictus* in L4 ($b$ ranging from 0 to 1, mean = 0.84, median = 1.00).

In the MMA beta-binomial logistic regression model, the most important covariate (Fig. S2) was the oviposition surface (present in ca. 90% of the selected models), followed by the logarithm of egg density (ca. 65%), and the proportion of *Ae. albopictus* in total L4 count (ca. 55%). The interaction term between the proportion of *Ae. albopictus* and the logarithm of egg density was rarely selected (20%).

The estimated model coefficients are shown in Table S5. In addition to the model intercept, the only significant coefficient was the oviposition surface (P = 0.003). Its value for the blotting paper strip category was negative (− 0.57), i. e., a lower survival was observed for L4 emerging from eggs collected on blotting paper strips, holding the other covariates constant.

**Figure 6:**
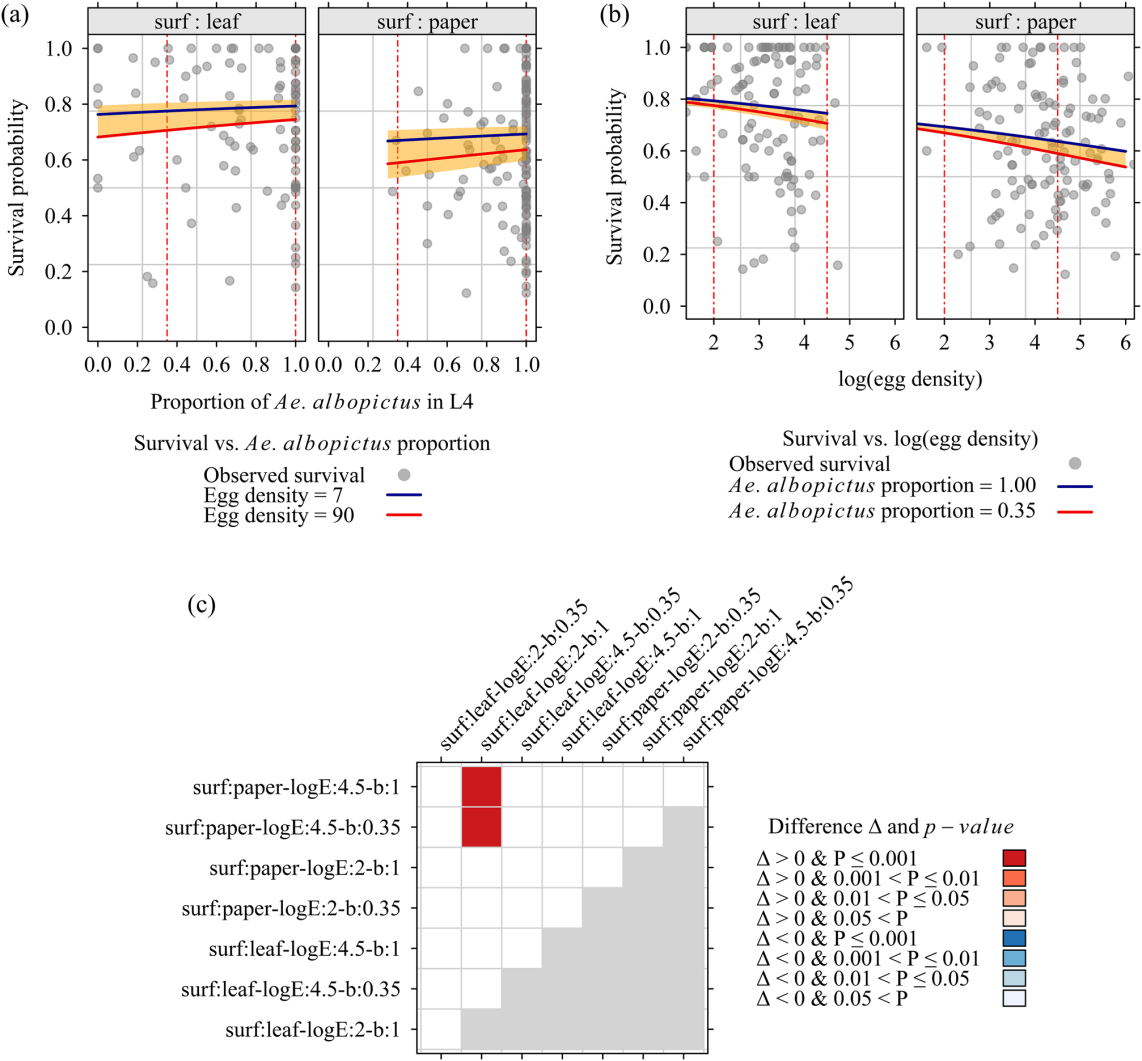
Fitted survival of fourth-instar larvae (L4) emerging from collected *Aedes* eggs by oviposition surface (**a**) according to the proportion of *Aedes albopictus*, holding the egg density constant (**b**) according to log(egg density), holding the proportion of *Aedes albopictus* constant. Vertical, red-dashed lines were drawn at the tested differences (**c**) pairwise tests for the differences d(*i*, *j*) in estimate (column *j*–row *i*. Column and row labels: surf oviposition surface: leaf (vacoa leaf) / paper (paper strip). logE logarithm of collected eggs (2.0/4.5); (**b**) proportion of *Aedes albopictus* in L4 (0.35/1.00). The orange-shaded band represents the range of fitted survival according to the observed range of covariates. Models were fitted with data collected during a field experiment in La Reunion, 2020 (256 ovitrap outcomes, 11,826 eggs).

To facilitate model interpretation, and to allow comparisons of interesting population means, we built a grid of predicted values, taking care to predict within the space defined by the observed covariates (Fig. 6).

The only significant differences were found between the patterns “*surf* = ‘leaf’ + *logE* = 2 + *b* = 1” (survival $\simeq$ 0.80) on the one hand, and “*surf* = ‘paper’ + *logE* = 4.5 + *b* = 0.35” (survival $\simeq$ 0.58), on the other hand (Fig. 6a,b for the survival, and Fig. 6c for the *p-values*).

In conclusion, L4 survival was lower when eggs were collected on a blotting paper strip (vs. a piece of vacoa leaf). However, the difference in survival was only significant at high egg densities.

At this stage, we can fully answer the question asked:

What is the best setting for ovitraps - including for the L4 laboratory-rearing step, to maximize the probability to detect Ae. aegypti and Ae. albopictus mosquitoes, and assess the relative apparent density of their populations?

1. Place the ovitraps in vacoa trees at man’s height, not on the ground, to allow the detection of *Ae. aegypti*.
2. Use a piece of vacoa leaf as the oviposition surface, not a blotting paper strip, to avoid high egg density associated with density dependent larval mortality.

## Discussion

### Data visualization and analysis

Visualization tools were developed for the purpose of this study—i.e. based on level plots^38,39^, to quickly identify meaningful P-value patterns in (otherwise cumbersome) matrices of *p-values*﻿ of tested differences in population means (Figs. 2b,d, 3b,d, 4b, 6c). Commonalities, as well as peculiarities of each mosquito’s ecology were thus easily identified. Such visualization tools, and underlying visualization rules, should deserve more attention for better communication between researchers and users of their findings.

### Trapping device and baiting

Our study provided yet another evidence of the efficiency of black plastic pots as ovitraps. Such devices are basic tools for public-health users and researchers in La Reunion. *Aedes albopictus* and *Ae. aegypti* both oviposit in man-made containers. The selection of black ovitraps used to monitor populations was based on their preference to oviposit in small black or dark water-holding containers resembling tree holes or rock cavities^34,35^.

The amount of water filling our oviposition traps, reached approximately 1/3 of the total height of the trap. It was very attractive for *Ae. albopictus*. Delatte et al.^26^ reported that an average depth of 6 cm of clear water was an optimal filling for *Ae. albopictus*. Only tap water was used throughout our study. It was the most efficient to collect eggs from *Ae. albopictus* in another study^35^.

### Organic matter in trap water

No strong preference was found for the trap location, or the addition of organic matter. This can be considered an adaptive behavior of females, that select various larval habitats relying on the higher competitiveness of their larvae. The same findings were made by Yoshioka et al.^40^, along with the evidence that the presence and density of con-specific larvae were the most important factors influencing site selection in a medium-to-low diet gradient.

The lack of effect of organic matter on the detection probability and m:apparent density of *Ae. aegypti* L4 was also reported by Wong et al.^41^ for which *Ae. aegypti* density was increased by the presence of conspecific larvae and pupae in the oviposition device, its size, and its exposure to sunlight.

Conversely, Hawley^42^ reported that the availability of organic matter and food was essential for the survival of *Ae. albopictus* larvae and for their adult size, all the more in natural vs. artificial containers. Females were likely to choose oviposition sites loaded with organic matter. Delatte et al.^26,43^ observed that oviposition sites presenting organic matter (medium to high levels) in shaded locations provided ideal habitats for larval and pupal development of *Ae. albopictus*. In addition, Daugherty et al.^44^ found that invertebrate carcasses favored larval development. Nevertheless, we did not find strong differences in apparent densities with the addition of organic matter (fish food: byproducts of fishes, mollusks and crustaceans) at a standardized dose. It might be interesting to increase the doses of organic matter to test possible increases in attractiveness.

### Trap position

Female mosquitoes select specific and appropriate habitats to lay their eggs, so that resource-mediated interactions are minimized, and offspring health is preserved^45–47^. In this study, female *Ae. aegypti* and *Ae. albopictus* had contrasted oviposition behaviors. The ovitrap height position—at the ground level or in the vacoa canopy, had a strong and positive effect on the probability to detect *Ae. aegypti* L4, as well as on the estimate of their apparent density. We are not aware of previous mentions of *Ae. aegypti* oviposition preferences for vacoa trees in La Reunion, as we observed during preliminary screening of the larval habitats (Figs. S3 and S4).

The strong preference of local *Ae. aegypti* populations for vacoa trees—and other natural oviposition sites, is probably an adaptation to their environment. As a matter of fact, water resources available at the basis of vacoa leaves are low (Fig. 1c). Consequently, so are the food resources and larval charge capacity associated with this breeding site. Local strains of *Ae. aegypti* have been coevolving much longer with vacoa trees – an endemic tree species from Mascareign Islands, and may thus be better adapted than *Ae. albopictus* mosquitoes which were only recently introduced in La Reunion, originating from southeastern Asia^23^. Therefore, they can compete with *Ae. albopictus* in this environment^24^. The main factor conditioning presence and abundance was different for the two species, corresponding to partially different niches, as shown by the striking difference in the pattern of pairwise-test matrices representations in Figs. 2c,d, and 3c,d. This is consistent with the higher *Ae. aegypti* density found in the vacoa tree canopy, where breeding sites are smaller, and more prone to desiccation, than at ground level. Indeed, previous studies reported local *Ae. aegypti* mosquitoes were more resistant to desiccation and high temperature than *Ae. albopictus*: a drier environment favors them^48,49^. Although this knowledge seems to be new to scientists, local inhabitants are well aware of the abundance of *Aedes* larvae in vacoa trees, as observed during the communication campaign of this study. Managing vacoa trees in private properties might thus be considered part of the integrated control of *Aedes* mosquitoes in La Reunion, taking into account the importance of these trees for other endemic species such as the Manapany day gecko *Phelsuma inexpectata*^50^.

### Oviposition surface and egg density

The proportion of L4 *Ae. aegypti* emerging from eggs collected in ovitraps depended on (i) the oviposition surface, and (ii) egg density. Ovitraps placed in vacoa trees—with vacoa leaves as the oviposition surface, were the most favorable to a high proportion (ca. 40% in our study). The actual egg ratio of *Ae. aegypti* was likely underestimated by the proportion of *Ae. aegypti* in L4 because of the higher mortality affecting their larvae when competing with *Ae. albopictus* larvae.

The better larval survival found for oviposition surfaces with lower egg density is not surprising. Braks et al.^51^ and Moore & Fisher^52^ showed the survival of *Ae. albopictus* and *Ae. aegypti* mosquitoes decreased as their density increased either for single or mixed populations. In addition to the direct depredation of *Ae. aegypti* larvae by *Ae. albopictus*, we also observed that during the laboratory-rearing step, the higher the egg density was, the longer the larval development time, and the smaller the larval size at the L4 stage, and the higher the mortality. This is consistent with the better survival observed with the vacoa-leaf oviposition surface, because of the lower egg density associated with this surface. However, the results of the beta-binomial logistic regression analysis (Table S5) provided evidence of a negative effect of the blotting paper on L4 survival, independent of the egg density (P = 0.002). This might be due to the direct, positive effects of specific compounds released in the trap water by vacoa leaves on *Aedes* larvae, or indirect effects through a microbiota-mediated impact. Further research would is needed to draw conclusions.

### Ecology and DV transmission

Females of *Ae. albopictus* mosquitoes lay their eggs in two types of habitat: natural sites, and artificial sites^23^. The two sites where we conducted our study were both in urban or peri-urban areas, the houses being only a few meters away from the nearest oviposition traps. Domestic and peridomestic potential breeding sites, such as wasted containers, flower cups, or used tires, are favorable oviposition sites for *Ae. albopictus*. Peridomestic and natural sites represent more random habitats, as they are more influenced by seasonal changes in climate and rainfall than domestic sites whose water supply is more dependent on anthropogenic activities^23^. Thus, in difficult conditions for *Ae. albopictus*, its competitive advantage over *Ae. aegypti* is mostly abolished. After the initial decline in the distribution of *Ae. aegypti* following the introduction of *Ae. albopictus* in La Reunion and the subsequent changes affecting the island (urbanization, anthropization of natural environments, malaria vector control campaigns), it seems likely that *Ae. aegypti* populations were able to survive in places where such natural larval habitats are present.

In a field experiment to compare different baiting schemes for adult traps—implemented in the same sites and vector season, we reported the same order of magnitude for the apparent densities of these populations, when adult traps were baited with CO_2_^37^, or when we set the ovitraps in vacoa canopy, with vacoa leaves for oviposition. The two studies were performed—by chance, during a large DV outbreak that affected Saint-Joseph and many other municipalities. Therefore, *Ae. aegypti* mosquitoes might play a role in DV transmission in such ecosystems.

## Conclusion

*Aedes* mosquitoes show a great ecological plasticity, and have a global distribution covering different ecosystems, at different altitudes and latitudes, and under a large range of environmental conditions. Our results extend the current knowledge on the ecology of *Ae. aegypti* and *Ae. albopictus*, by quantifying the preferences of female mosquitoes for three oviposition parameters.

This study of oviposition preferences, as well as the determination of the biological and ecological factors regulating the natural *Aedes* populations in La Reunion, will be useful for the development of sound monitoring and control strategies, especially for genetic control methods. It also shows that local knowledge on *Aedes* ecology may be accounted for when designing integrated control strategies, which will in turn facilitate their appropriation by local populations.

In addition to the specific results presented here, special attention was paid to the whole methodological package to monitor *Aedes* populations, particularly data analysis. Methods and tools for monitoring *Aedes* populations, such as this ovitrap assessment, are selected and codeveloped together with local and regional public-health practitioners, to identify and address their actual needs. In turn, regional meetings and training courses are being organized and will be extended as new results are available.

Indeed, past events highlighted the utmost importance of being able and ready to prevent, detect, mitigate, and monitor disease outbreaks linked to *Aedes* mosquito vectors, such as the DV—as well as chikungunya, Zika, or any other / new emerging mosquito-borne virus.

## Methods

### Biological data

#### Harvesting and processing mosquito eggs

Each day, all oviposition surfaces were collected from the ovitraps. In addition, their water was filtered to collect possible *Ae. aegypti* eggs as these mosquitoes also lay their eggs on the water surface of breeding sites^53^.

The collected oviposition surfaces were placed in a controlled-atmosphere chamber (air temperature: 28 ± 2 °C, relative humidity: 70 ± 10% RH, and photoperiod: 12:12 h (light:dark) at Cirad laboratory, Saint-Pierre (La Reunion) for one week, to dry the oviposition surfaces.

#### Rearing mosquito larvae

Eggs were counted and placed in 400-mL plastic containers covered with mosquito netting, and filled with 250 mL of tap water. Larvae were grown in the same greenhouse. They were fed crushed dry cat food (Friskies Adults)^54^. As they grew, they were fed with an increasing amount of food. This amount was kept low to avoid spurious bacterial growth—a possible cause of larval death^32^. It was computed on the basis of four mg/larvae throughout the rearing stage, with 10% at *d*_0_ + 1, 45% at *d*_0_ + 2, and 45% at *d*_0_ + 4 (*d*_0_: day when eggs were counted and flooded). Larvae reaching the fourth instar (L4) at 4–5 days were stored in a freezer (+ 4 °C), identified using an ad-hoc procedure^55^, and counted using a binocular microscope. All collected data were compiled in a database.

### Data analysis

Three questions were addressed in this field experiment—and consecutively, three different datasets were defined:

- 1. What is the best trapping setting for ovitraps to maximize the probability of detecting *Ae. aegypti* (and *Ae. albopictus* as well), and assess its apparent density?
- 2. What is the best trapping setting for ovitraps to provide accurate estimates for the proportion of mosquitoes from both species?
- 3. What is the best laboratory-rearing setting to maximize the probability of survival of immature-stage up L4?

#### Question-specific models

##### Detection probability and apparent density

We chose a hurdle model^56^ made of two submodels that were jointly fitted: (i) a logistic Bernoulli model for the presence data (detection probability), and a zero-truncated negative binomial model for the apparent-density counts. The same covariates, defined above, were used to model the detection probability and the apparent density.

##### The proportion of *Ae. aegypti* in total L4 count

The previous steps of the analysis provided evidence that *Ae. aegypti* rarely goes to the ground. Therefore, we discarded data collected at the ground level, and compared the proportions of *Ae. aegypti* according to the oviposition surface, and the addition of organic matter in trap water (covariates). We did not use models: we used the proportion of *Ae. aegypti* in the original dataset as an estimate of population means, and used the procedure described below to estimate its credible intervals and test proportion differences between groups defined by the covariates.

##### Survival of L4 at the laboratory-rearing stage

The survival of L4 was the ratio $L/E$, where $E$ was the number of collected eggs, and $L$ the overall number of surviving L4 on the day the were counted. We discarded records with $E=0$. We considered three covariates (1) the proportion *b* of *Ae. albopictus* in the final L4 count (ranging from 0 to 1), included to assess the difference in survival between *Ae. albopictus* and *Ae. aegypti*, (2) the logarithm of the egg count (*logE*, ranging from 0.69 for $E=2$, to 6.43 for $E=619$)) to assess the possible density-dependent effect on larvae mortality; (3) the oviposition surface (*surf*: blotting paper strip vs. piece of vacoa leaf) to assess the effect of oviposition surface. We used a beta-binomial logistic regression model to analyze survival.

### Estimates, credible intervals, and tests

#### Population means

In the analyzed datasets, the outcome (L4 count, or proportion) showed a large variance. Beyond the usual overdispersion with respect to Poisson or binomial distributions, met in many studies using insect counts^57^, this large variance was related to the peculiarities of *Aedes* mosquito ecology. At the single exception of one count in the apparent density dataset which was spuriously influential in model coefficients—and thus, predicted population means ($m=45$ *Ae. aegypti* L4, ovitrap on the ground), we did not discard the corresponding data because they are an important feature of *Aedes* ecology. Therefore, we did not expect to select a single best model adapted to an unrealistic “average” situation.

Instead, we adopted a multimodel averaging (MMA) approach, which is well suited when there is not a single plausible model with respect to the available data and model coefficients to be estimated^58^. A “full” model was defined, representing the most complex structure expected in the dataset. For the detection probability, and the apparent density datasets, this was the additive model with the three covariates studied (oviposition surface, height position in the vacoa tree, and addition of organic matter). For the survival of L4, the full model had four terms: the oviposition surface, the logarithm of the initial egg count, the proportion of *Ae. albopictus* mosquitoes in the final L4 count, and the interaction between these two covariates.

From each full model, the set of all possible submodels was defined. For the survival model, when the interaction term was considered, we also included the main effects involved in the interaction. For each dataset, all the models were fitted using a maximum-likelihood method. Then, they were ranked according to the AICc, i.e., the small-sample version of the Akaike information criterion^59^. The AICc difference between consecutive ranked models was computed, and then divided by the AICc difference between the first and last models. This quantity is the Akaike weight^60^. It was used as an indicator of relative model plausibility, and to compute the weighted means of the fitted values from each compared model, i.e., multimodel averaging^60^.

#### Credible intervals and tests

To estimate the 95% credible intervals of the statistics of interest (differences between population means), we used a bootstrap resampling procedure of the observed dataset^61^. The combination of sites and dates (two sites, and four dates by replicate, and three replicates) was taken as the 24 resampling units of four trapping sessions each (i.e., the four dates). A random sample of these units was drawn with replacement in the initial data set, with the same size in sampling units, and 1000 replicates. It was used to compute the simulated means and their differences.

- The 2.5% and 97.5% quantiles of the empirical distribution of each simulated mean were used as the 95% credible interval of population means.
- To assess the significance of differences in population means corresponding to ovitrap settings, or rearing conditions, we computed them from the original dataset. The null hypothesis to be tested for each difference was that its credible interval included zero. To implement the test, we computed each difference for the series of bootstrapped datasets. Then, using a basic search procedure, we looked for the smallest $\alpha$ value for each difference (series of B values)—starting from $\alpha =0$, so that the credible interval defined by the two quantiles $\alpha$/2 and 1 − $\alpha$/2 did not include zero (actually, we tested whether the product of these quantiles was > 0).

#### P-value adjustment for multiple comparisons, and visualization

In both analyses (detection/apparent density, and survival), we compared height population means. Therefore, we had to assess 8 * 7 / 2 = 28 differences, thus encountering the issue of multiple comparisons using the same sample—possibly leading to spuriously small *p-values* and drawing erroneous conclusions (i.e., rejecting the null hypothesis too often). To address this situation, we adopted the Holm procedure to obtain *p-values* adjusted for multiple comparisons^62^.

To facilitate the interpretation of all these *p-values*, and the identification of possible specific ecological patterns, we plotted them using level plots of P-value matrices. Each cell of the matrix contained a probability code for the *p-value* category associated with a difference $\Delta$ (column *j*–row *i*) between the population mean indexed by column *j*, and the population mean indexed by row *i*. The color associated with ${\Delta }_{i,j}$ was defined by two criteria:

- 1. the sign of ${\Delta }_{i,j}$: red if ${\Delta }_{i,j}>0$, blue if ${\Delta }_{i,j}<0$;
- 2. a code (1, 2, 3, or 4) for the *p-value, *defined by the following probability breaks: 0, 0.001, 0.01, 0.05, and 1. The code was associated with a shade of red or blue—according to the sign of ${\Delta }_{i,j}$: the smaller the code (i.e., the smaller the *p-value*, the darker the shade). Because the matrix was symmetric, with a diagonal of zero, we only represented its upper side (above the diagonal), omitting the diagonal.

In addition, matrix columns and rows were ordered according to the importance of covariates involved in the tested differences, to facilitate interpretation of the results.

The R software and computing environment^63^ were used for data analysis. In addition, we used add-on packages for the most specialized features: pscl for hurdle models^56^, aods3 for beta-binomial logistic regression^64^, and MuMIn for multi-model averaging and inference^65^, implementing the information-theoretical approach and methods described in Burnham & Anderson^60^.

## Supplementary Information

Supplementary Information.

Supplementary Information.

## Data Availability

The data—and R code needed to run the data analysis, are available in a zip file (ovitrap.zip) found in the Supplementary Information material. The master R code is gathered in an R Markdown document^66^ located at the root of the unzipped file. Data are available as a csv file located in the subfolder called data. Most of the statistical work is done by R functions automatically sourced in the running R session from an external file called functions.R. The list of add-on R packages needed to run the data analysis—code to install and update them, are available in the file packages.R, also sourced in the R session.

## References

[1] Yang, X., Quam, M. B., Zhang, T. & Sang, S. Global burden for dengue and the evolving pattern in the past 30 years. *J. Travel Med.* **28**, taab146 (2021). 10.1093/jtm/taab146 34510205

[2] Simmons CP, Farrar JJ, van Vinh Chau N, Wills B (2012). Dengue. N. Engl. J. Med..

[3] Brady OJ (2012). Refining the global spatial limits of dengue virus transmission by evidence-based consensus. PLoS Negl. Trop. Dis..

[4] Morin CW, Comrie AC, Ernst K (2013). Climate and dengue transmission: Evidence and implications. Environ. Health Perspect..

[5] Brady, O. J. *et al.* Global temperature constraints on Aedes aegypti and Ae. albopictus persistence and competence for dengue virus transmission. *Parasites Vectors* **7**, 338 (2014). 10.1186/1756-3305-7-338 PMC4148136 25052008

[6] Betanzos-Reyes, Á. F. *et al.* Association of dengue fever with Aedes spp. abundance and climatological effects. *Salud Pública de México* **60**, 12 (2017). 10.21149/8141 29689652

[7] WHO. Dengue and severe dengue. (2022).

[8] Gubler DJ (1998). Dengue and dengue hemorrhagic fever. Clin. Microbiol. Rev..

[9] Delatte H (2008). Aedes albopictus, vecteur des virus du chikungunya et de la dengue à La Réunion : biologie et contrôle. Parasite.

[10] Kles V, Michault A, Rodhain F, Mevel F, Chastel C (1994). A serological survey regarding Flaviviridae infections on the island of Reunion (1971–1989). Bull. Soc. Pathol. Exot..

[11] Pierre, V. *et al.* Epidémie de dengue 1 à la Réunion en 2004. *Journal de Veille Sanitaire* (2005).

[12] Vincent, M. *et al.* From the threat to the large outbreak: dengue on Reunion Island, 2015 to 2018. *Eurosurveillance* **24**, (2019). 10.2807/1560-7917.ES.2019.24.47.1900346 PMC6885751 31771702

[13] Cellule Santé Publique France en Région, ARS. *Situation de la dengue à La Réunion au 15 décembre 2020*. https://www.lareunion.ars.sante.fr/avec-le-retour-de-lete-agissons-des-maintenant-contre-la-dengue (2020).

[14] Agence Régionale de Santé. *Communiqué de presse: dengue à La Réunion. Situation au 28 juillet 2021*. https://www.lareunion.ars.sante.fr/system/files/2021-07/2021-07-28-Dengue-Situation à La Réunion_0.pdf (2021).

[15] Hafsia S (2022). Overview of dengue outbreaks in the southwestern Indian Ocean and analysis of factors involved in the shift toward endemicity in Reunion Island: A systematic review. PLoS Negl. Trop. Dis..

[16] Reiter P, Fontenille D, Paupy C (2006). Aedes albopictus as an epidemic vector of chikungunya virus: Another emerging problem?. Lancet. Infect. Dis.

[17] Njenga MK (2008). Tracking epidemic Chikungunya virus into the Indian Ocean from East Africa. J. Gen. Virol..

[18] Soumahoro M-K (2011). The Chikungunya epidemic on La Réunion Island in 2005–2006: a cost-of-illness study. PLoS Negl. Trop. Dis..

[19] Larrieu, S., Balleydier, E., Renault, P., Baville, M. & Filleul, L. [Epidemiological surveillance du chikungunya on Reunion Island from 2005 to 2011]. *Médecine tropicale : Revue du Corps de Santé colonial* **72 Spec No**, 38–42 (2012). 22693926

[20] Soghigian J (2020). Genetic evidence for the origin of Aedes aegypti, the yellow fever mosquito, in the southwestern Indian Ocean. Mol. Ecol..

[21] Kotsakiozi P (2018). Population structure of a vector of human diseases: Aedes aegypti in its ancestral range Africa. Ecol. Evol..

[22] MacGregor ME (1924). Aedes (Stegomyia) mascarensis, MacGregor: A new Mosquito from Mauritius. Bull. Entomol. Res..

[23] Salvan M, Mouchet J (1994). Aedes albopictus et Aedes aegypti à l’Ile de La Réunion. Ann. Soc. Belg. Med. Trop..

[24] Bagny L, Delatte H, Quilici S, Fontenille D (2009). Progressive Decrease in Aedes aegypti distribution in Reunion Island since the 1900s. J. Med. Entomol..

[25] Le Vassal JJ (1907). paludisme à l’Ile de La Réunion. Per Gli Stud Della Maria.

[26] Delatte H (2008). Geographic distribution and developmental sites of Aedes albopictus (Diptera: Culicidae) during a chikungunya epidemic event. Vector-Borne Zoon. Dis..

[27] Hamon J (1953). Etudes biologique et systématique des Culicinae de l’Ile de La Réunion. Mem. Inst. Scient. Madagascar.

[28] Bouyer J, Lefrançois T (2014). Boosting the sterile insect technique to control mosquitoes. Trends Parasitol..

[29] Haramboure M (2020). Modelling the control of Aedes albopictus mosquitoes based on sterile males release techniques in a tropical environment. Ecol. Model..

[30] Bouyer J, Yamada H, Pereira R, Bourtzis K, Vreysen MJB (2020). Phased conditional approach for mosquito management using sterile insect technique. Trends Parasitol..

[31] Bouyer J, Vreysen MJB (2020). Yes, irradiated sterile male mosquitoes can be sexually competitive!. Trends Parasitol..

[32] Organization, W. H. Guidelines for laboratory and field testing of mosquito larvicides. WHO/CDS/WHOPES/GCDPP/2005.13 (2005).

[33] World Health Organization and Special Programme for Research and Training in Tropical Diseases and World Health Organization. Department of Control of Neglected Tropical Diseases and World Health Organization. Epidemic and Pandemic Alert. *Dengue: Guidelines for diagnosis, treatment, prevention and control*. (World Health Organization, 2009).

[34] Yap HH (1975). Preliminary report on the color preference for oviposition by Aedes albopictus (Skuse) in the field. Southeast Asian J. Trop. Med. Public Health.

[35] Yap HH, Lee CY, Chong NL, Foo AES, Lim MP (1995). Oviposition site preference of Aedes albopictus in the laboratory. J. Am. Mosquito Control Assoc. Mosquito News.

[36] Marin G, Mahiba B, Arivoli S, Tennyson S (2020). Does colour of ovitrap influence the ovipositional preference of Aedes aegypti Linnaeus 1762 (Diptera: Culicidae). Int. J. Mosq. Res.

[37] Claudel, I. *et al.* To bait or not to bait? Optimizing the use of adult mosquito traps for monitoring arbovirus vector populations in La Réunion Island. (2022). 10.21203/rs.3.rs-1798972/v1.

[38] Cleveland, W. S. *Visualizing data*. (Hobart press, 1993).

[39] Lamigueiro, Ó. P. *Displaying time series, spatial, and space-time data with R*. (Chapman; Hall/CRC, 2018).

[40] Yoshioka, M. *et al.* Diet and density dependent competition affect larval performance and oviposition site selection in the mosquito species Aedes albopictus (Diptera: Culicidae). *Parasites Vectors* **5**, (2012). 10.1186/1756-3305-5-225 PMC3481443 23044004

[41] Wong J, Stoddard ST, Astete H, Morrison AC, Scott TW (2011). Oviposition site selection by the dengue vector Aedes aegypti and its implications for dengue control. PLoS Negl. Trop. Dis..

[42] Hawley, W. A. The biology of aedes albopictus. *J. Am. Mosquito Control Assoc. Suppl* **1**, 1–39 (1988). 3068349

[43] Delatte H, Gimonneau G, Triboire A, Fontenille D (2009). Influence of temperature on immature development, survival, longevity, fecundity, and gonotrophic cycles of Aedes albopictus, vector of Chikungunya and dengue in the Indian Ocean. J. Med. Entomol..

[44] Daugherty MP, Alto BW, Juliano SA (2000). Invertebrate carcasses as a resource for competing Aedes albopictus and Aedes aegypti (Diptera: Culicidae). J. Med. Entomol..

[45] Papaj, D. R. & Rausher, M. D. Individual variation in host location by phytophagous insects. *Herbivorous Insects: Host seeking behavior and mechanisms* 77–127 (1983).

[46] Valladares G, Lawton JH (1991). Host-plant selection in the holly leaf-miner: Does mother know best?. J. Anim. Ecol..

[47] Ellis AM (2008). Incorporating density dependence into the oviposition preference-offspring performance hypothesis. J. Anim. Ecol..

[48] Juliano, S. A., OMeara, G. F., Morrill, J. R. & Cutwa, M. M. Desiccation and thermal tolerance of eggs and the coexistence of competing mosquitoes. *Oecologia* **130**, 458–469 (2002). 10.1007/s004420100811 PMC2944657 20871747

[49] Costanzo KS, Kesavaraju B, Juliano SA (2005). Condition-specific competion in container mosquitoes: The role of non-competing life-history stages. Ecology.

[50] Sanchez, M. & Probst, J.-M. Distribution and conservation status of the Manapany day gecko, Phelsuma inexpectata MERTENS, 1966, an endemic threatened reptile from Réunion Island (Squamata: Gekkonidae). *Cahiers scientifiques de l’océan Indien occidental* **2**, (2011).

[51] Braks MAH, Honório NA, Lounibos LP, De-Oliveira RL, Juliano SA (2004). Interspecific competition between two invasive species of container mosquitoes, Aedes aegypti and Aedes albopictus (Diptera: Culicidae), in Brazil. Ann. Entomol. Soc. Am..

[52] Moore, C. G. & Fisher, B. R. Competition in mosquitoes.1 Density and species ratio effects on growth, mortality, fecundity, and production of growth retardant2. *Ann. Entomol. Soc. Am.* **62**, 1325–1331 (1969). 10.1093/aesa/62.6.1325 5374170

[53] Madeira NG, Macharelli CA, Carvalho LR (2002). Variation of the Oviposition Preferences of Aedes aegypti in Function of Substratum and Humidity. Mem. Inst. Oswaldo Cruz.

[54] Bellini, R. *et al.* Use of the sterile insect technique against Aedes albopictus in Italy: first results of a pilot trial. in *Area-wide control of insect pests* 505–515 (Springer, 2007).

[55] Boussès P, Dehecq JS, Brengues C, Fontenille D (2013). Inventaire actualisé des moustiques (Diptera : Culicidae) de l’île de La Réunion, océan Indien. Bulletin de la Société de pathologie exotique.

[56] Zeileis, A., Kleiber, C. & Jackman, S. Regression models for count data in R. *J. Stat. Softw.* **27**, (2008).

[57] Sileshi G (2006). Selecting the right statistical model for analysis of insect count data by using information theoretic measures. Bull. Entomol. Res..

[58] Guthery FS, Burnham KP, Anderson DR (2003). Model selection and multimodel inference: A practical information-theoretic approach. J. Wildl. Manag..

[59] Hurvich CM, Tsai C-L (1995). Model selection for extended quasi-likelihood models in small samples. Biometrics.

[60] Burnham, K. P. & Anderson, D. R. *Model selection and multimodel inference: a practical information-theoretic approach*. 496 (Springer-Verlag, 2002).

[61] Manly, B. F. J. *Randomization, bootstrap and Monte Carlo methods in biology*. 399 (CRC Press / Chapman & Hall, 2006). 10.1201/9781315273075.

[62] Holm, S. A simple sequentially rejective multiple test procedure. *Scand. J. Stat.* 65–70 (1979).

[63] R Core Team. *R: A Language and Environment for Statistical Computing*. (R Foundation for Statistical Computing, 2022).

[64] Lesnoff, M. & Lancelot, R. *aods3: analysis of overdispersed data using S3 methods*. (2018).

[65] Barton, K. *MuMIn: Multi-Model Inference*. (2022).

[66] Xie, Y., Dervieux, C. & Riederer, E. *R Markdown Cookbook*. (Chapman; Hall/CRC, 2020). 10.1201/9781003097471.

