# Supplementary material for: Optimization of oviposition trap settings to monitor populations of Aedes mosquitoes, vectors of arboviruses in La Reunion
Source: Sci Rep. 2022 Nov 2;12:18450. doi: 10.1038/s41598-022-23137-5 (PMC9630495; doi:10.1038/s41598-022-23137-5)
Supplement: Supplementary file 1 — Supplementary Information. [file 41598_2022_23137_MOESM1_ESM.docx]

Optimizing the use of oviposition traps to monitor *Aedes* mosquito populations, vectors of arboviruses in La Reunion

Supplementary Information

Ronan Brouazin^1,a^, Iris Claudel^1,a^, Renaud Lancelot^2,3^*, Guillaume Dupuy^4^, Louis-Clément Gouagna^5^, Marlène Dupraz^2,3^, Thierry Baldet^2,3^, Jérémy Bouyer^2,6^*

^1^ UMR Mivegec (Maladies Infectieuses et Vecteurs: Écologie, Génétique, Évolution et Contrôle), IRD-CNRS-Univ. Montpellier, 97410 Saint-Pierre, La Réunion

^2^ UMR Astre (Animals, Health, Territories, Risks, Ecosystems), Cirad, Inrae, Univ. Montpellier, 34398 Montpellier, France

^3^ Cirad, UMR Astre, 97491 Sainte Clotilde, La Réunion

^4^ ARS Réunion, Service de Lutte Anti-Vectorielle, Saint-Denis, La Réunion

^5^ UMR Mivegec, 34394 Montpellier, France

^6^ Insect Pest Control Laboratory, Joint FAO/IAEA Programme of Nuclear Techniques in Food and Agriculture, IAEA Vienna, Wagramer Strasse 5, 1400 Vienna, Austria

^a^ These authors contributed equally to the work

* Corresponding author: Jérémy Bouyer [jeremy.bouyer@cirad.fr](mailto:jeremy.bouyer@cirad.fr)

# 1 Figures


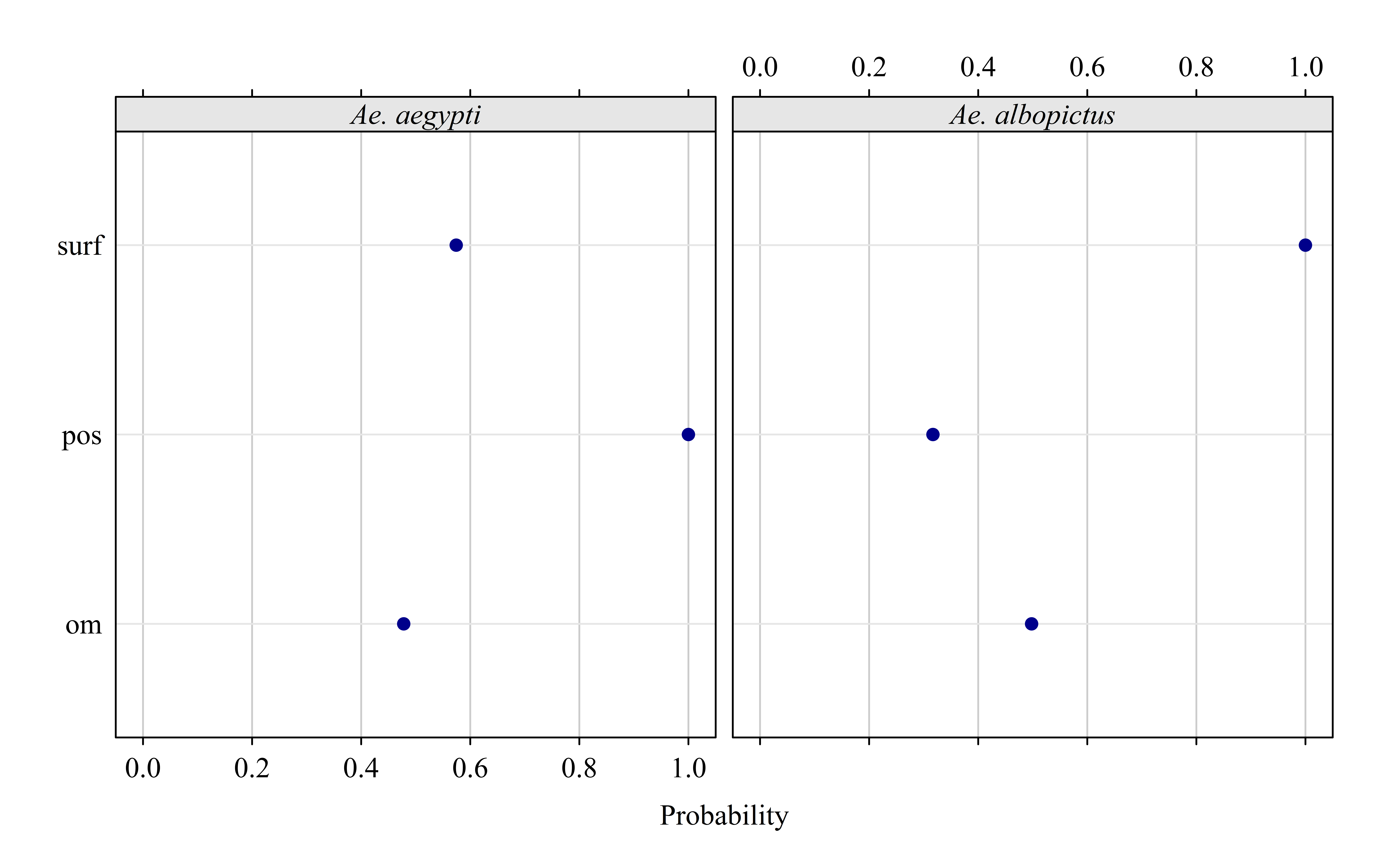


Fig. S1. Probability for a given covariate to be present in a model kept for multi-model averaging of daily apparent density of Aedes fourth-instar larvae, using data collected during a field experiment in La Reunion, 2020. Codes for ovitrap covariates: surf oviposition surface, pos height position in vacoa trees, om organic matter added to trap water.


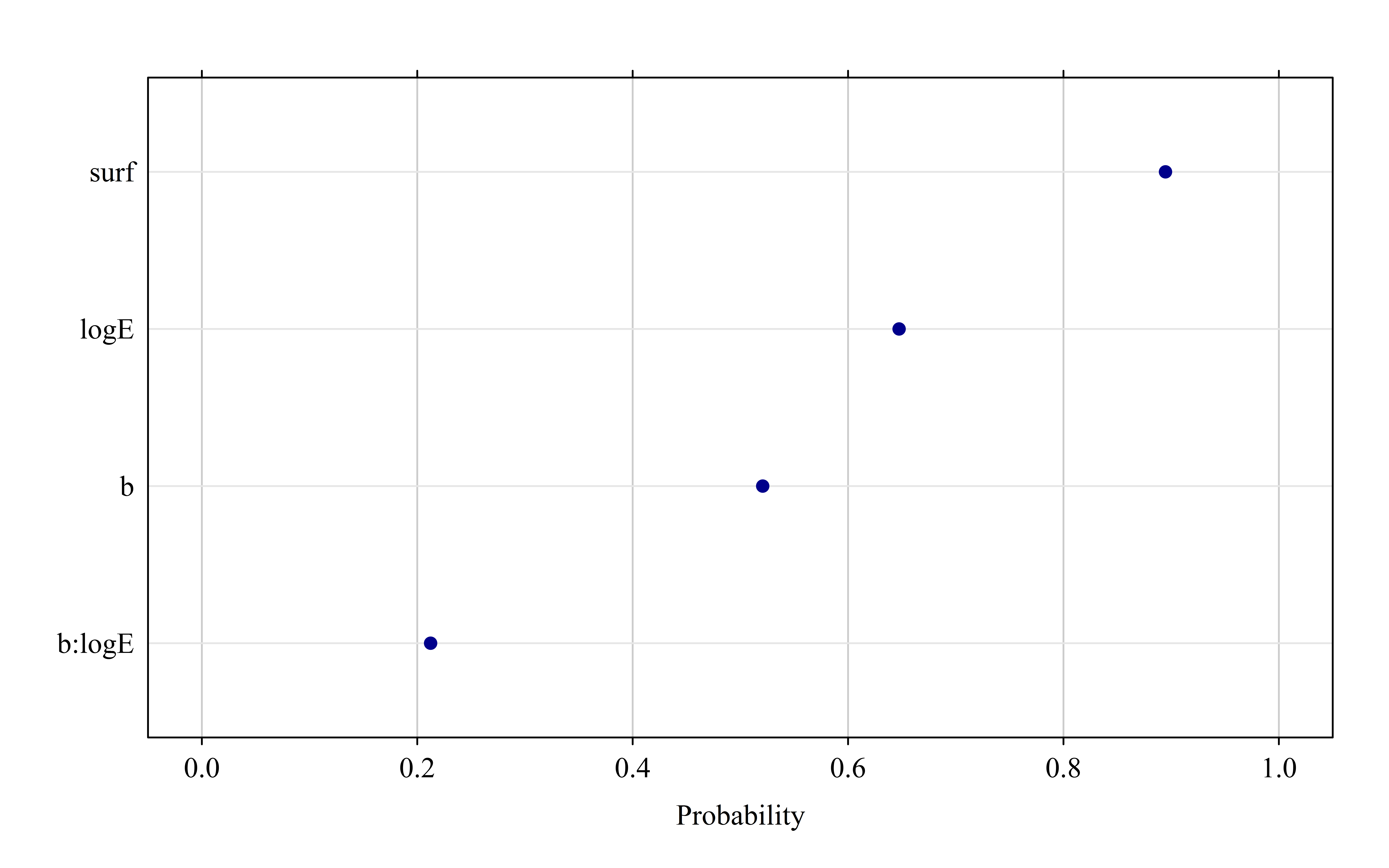


Fig. S2. Probability for a given covariate, to be present in a model kept for multi-model averaging of fourth-instar larvae survival from Aedes eggs collected during a field experiment in La Reunion, 2020. Codes for ovitrap covariates: surf oviposition surface, pos height position in vacoa trees, om organic matter added to trap water.

# 2 Tables

| Table S1. Daily apparent density of Ae. aegypti fourth-instar larvae estimated with data collected during a field experiment in La Reunion, 2020. | | | | |
| --- | --- | --- | --- | --- |
|  | | Fitted mean and 95% bootstrap credible interval (1,000 replicates) | | |
|  | Observed mean | Estimate | Lower limit | Upper limit |
| ****Trap on tree canopy**** | | | | |
| Oviposition surface: vacoa leaf | | | | |
| No organic matter added to trap water | 9.0 | 9.7 | 6.8 | 13.1 |
| No organic matter added to trap water | 8.5 | 8.2 | 5.4 | 11.5 |
| Oviposition surface: paper strip | | | | |
| No organic matter added to trap water | 12.4 | 10.9 | 7.4 | 16.6 |
| No organic matter added to trap water | 8.0 | 9.2 | 5.7 | 13.6 |
| ****Trap on the ground**** | | | | |
| Oviposition surface: vacoa leaf | | | | |
| No organic matter added to trap water | 0.3 | 0.5 | 0.1 | 1.3 |
| No organic matter added to trap water | 0.4 | 0.4 | 0.1 | 1.0 |
| Oviposition surface: paper strip | | | | |
| No organic matter added to trap water | 1.1 | 0.5 | 0.1 | 1.2 |
| No organic matter added to trap water | 0.0 | 0.4 | 0.1 | 0.9 |

| Table S2. Daily apparent density of Ae. albopictus fourth-instar larvae estimated with data collected during a field experiment in La Reunion, 2020. | | | | |
| --- | --- | --- | --- | --- |
|  | | Fitted mean and 95% bootstrap credible interval (1,000 replicates) | | |
|  | Observed mean | Estimate | Lower limit | Upper limit |
| ****Oviposition surface: vacoa leaf**** | | | | |
| Trap in tree canopy | | | | |
| Organic matter added to trap water | 14.2 | 14.6 | 11.3 | 18.5 |
| No organic matter added to trap water | 13.7 | 13.6 | 10.2 | 17.3 |
| Trap on the ground | | | | |
| Organic matter added to trap water | 15.1 | 14.2 | 10.9 | 17.9 |
| No organic matter added to trap water | 12.7 | 13.3 | 9.9 | 16.8 |
| ****Oviposition surface: paper strip**** | | | | |
| Trap in tree canopy | | | | |
| Organic matter added to trap water | 57.2 | 53.2 | 38.9 | 68.9 |
| No organic matter added to trap water | 48.1 | 48.6 | 35.6 | 63.5 |
| Trap on the ground | | | | |
| Organic matter added to trap water | 53.4 | 52.3 | 38.8 | 68.1 |
| No organic matter added to trap water | 43.4 | 47.8 | 35.7 | 61.1 |

| Table S3. Full multi-hurdle model averaged coefficients of Ae. aegypti fourth-instar larvae apparent density estimated with data collected during a field experiment in La Reunion, 2020. The model was made of two sub-models: (i) a zero-count Bernoulli logistic regression, and a negative-binomial zero-truncated count regression which were jointly fitted using a maximum-likelihood method. Codes for ovitrap covariates: surf oviposition surface, pos height position in vacoa trees, om organic matter added to trap water. | | | | |
| --- | --- | --- | --- | --- |
|  | Estimate | Std. Error | z value | Pr(>\|z\|) |
| Zero-count Bernoulli logistic regression | | | | |
| (intercept) | 1.043 | 0.284 | 3.675 | 0.000 |
| pos: ground | -3.380 | 0.386 | 8.756 | 0.000 |
| surf; paper | -0.215 | 0.335 | 0.641 | 0.521 |
| om: none | -0.057 | 0.190 | 0.303 | 0.762 |
| Zero-truncated negative-binomial regression | | | | |
| log(theta) | 0.180 | 0.203 | 0.887 | 0.375 |
| (intercept) | 2.528 | 0.146 | 17.306 | 0.000 |
| pos: ground | -2.116 | 0.420 | 5.036 | 0.000 |
| surf: paper | 0.098 | 0.173 | 0.568 | 0.570 |
| om: none | -0.034 | 0.110 | 0.305 | 0.761 |

| Table S4. Full multi-hurdle model averaged coefficients of Ae. albopictus fourth-instar larvae apparent density estimated with data collected during a field experiment in La Reunion, 2020. The model was made of two sub-models: (i) a zero-count Bernoulli logistic regression, and a negative-binomial zero-truncated count regression which were jointly fitted using a maximum-likelihood method. Codes for ovitrap covariates: surf oviposition surface, pos height position in vacoa trees, om organic matter added to trap water. | | | | |
| --- | --- | --- | --- | --- |
|  | Estimate | Std. Error | z value | Pr(>\|z\|) |
| Zero-count Bernoulli logistic regression | | | | |
| (intercept) | 1.396 | 0.267 | 5.236 | 0.000 |
| surf: paper | 1.597 | 0.474 | 3.370 | 0.001 |
| om: none | 0.075 | 0.249 | 0.301 | 0.763 |
| pos: ground | -0.031 | 0.165 | 0.190 | 0.850 |
| Zero-truncated negative-binomial regression | | | | |
| log(theta) | 0.257 | 0.107 | 2.404 | 0.016 |
| (intercept) | 2.847 | 0.109 | 26.140 | 0.000 |
| surf: paper | 1.171 | 0.121 | 9.660 | 0.000 |
| om: none | -0.060 | 0.110 | 0.542 | 0.588 |
| pos: ground | -0.006 | 0.047 | 0.138 | 0.890 |

| Table S5. Full multi-model averaged coefficients of fourth-instar larvae survival estimated with data collected during a field experiment in La Reunion, 2020. The averaged models were beta-binomial regressions of the proportion of surviving larvae among the initial egg count. Codes for ovitrap covariates: logE log-number of collected Aedes eggs; b proportion or Aedes albopictus in fourth-instar larvae; surf oviposition surface | | | | |
| --- | --- | --- | --- | --- |
|  | Estimate | Std. Error | z value | Pr(>\|z\|) |
| (intercept) | 1.296 | 0.382 | 3.394 | 0.001 |
| logE | -0.071 | 0.091 | 0.784 | 0.433 |
| surf: paper | -0.569 | 0.187 | 3.045 | 0.002 |
| b | 0.135 | 0.263 | 0.514 | 0.607 |

**3 Preliminary surveys on the ecology of *Aedes aegypti* in the study area**

Before starting the study, we implemented preliminary surveys to better understand the ecology of *Ae. aegypti* in the study area.

## 3.1 Screening larval habitats

Stipes of vacoa trees (*Pandanus edulis*) were the only natural mosquito breeding sites harboring *Aedes aegypti* larvae. Conversely, ponds and stones holes only harbored larvae from other mosquito species (fig. S3).


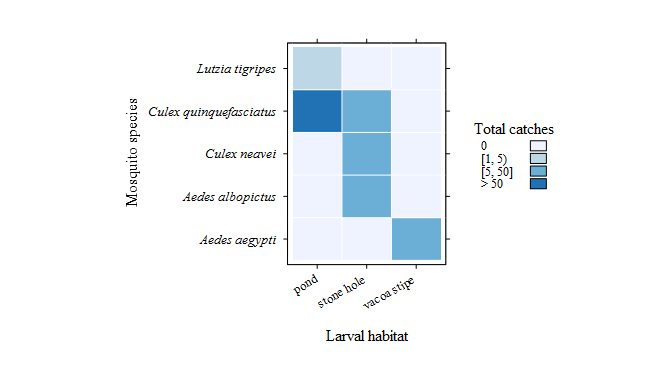


Fig. S3. Results of a screening survey (54 trapping outcome) to identify Aedes mosquito breeding sites in La Reunion, 2020.

## 3.2 Comparison of ovitrap settings

We also tested:

- stone ovitraps with various oviposition supports, baits and at various height positions,
- plastic ovitraps using a piece of vacoa leaf as an oviposition support and set in the canopy.

Only the latter ovitraps allowed collecting *Ae. aegypti* eggs, hence the design developed for this study.


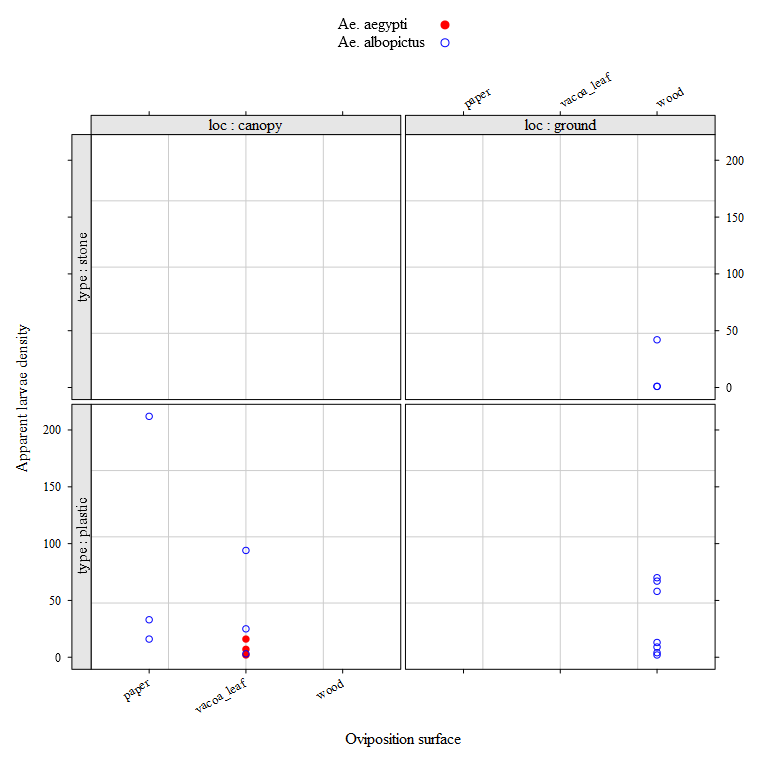


Fig. S4. Results of a screening survey (54 trapping outcome) to identify the ovitraps and settings allowing catches of Ae. albopictus and Ae. Aegypti mosquitoes in La Reunion, 2020.
