# Supplementary figures and images for: Optimization of oviposition trap settings to monitor populations of Aedes mosquitoes, vectors of arboviruses in La Reunion
Source: Sci Rep. 2022 Nov 2;12:18450. doi: 10.1038/s41598-022-23137-5 (PMC9630495; doi:10.1038/s41598-022-23137-5)

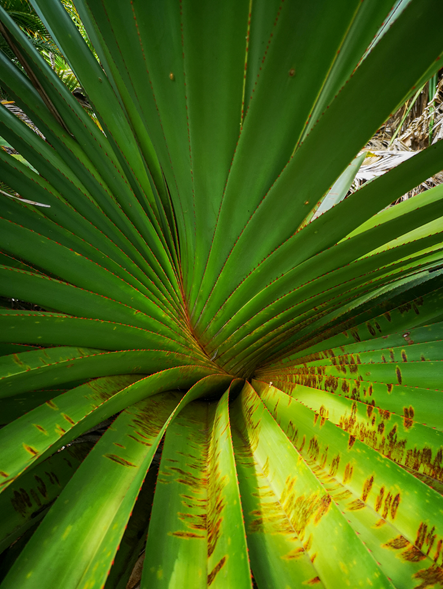

Supplement: Supplementary file 2 — Supplementary Information. [file 41598_2022_23137_MOESM2_ESM.zip › img/fig1.png]
